# Global Aromatic Ring Currents in Neutral Porphyrin Nanobelts

**DOI:** 10.1021/acsnano.4c14100

**Published:** 2024-12-31

**Authors:** Marco Vitek, Jie-Ren Deng, Harry L. Anderson, Igor Rončević

**Affiliations:** † Chemistry Research Laboratory, Department of Chemistry, University of Oxford, Oxford OX1 3TA, U.K.; ‡ Institute of Organic Chemistry and Biochemistry of the Czech Academy of Sciences, Flemingovo nám. 542/2, Prague 6 160 00, Czechia; § Department of Chemistry, The University of Manchester, Oxford Road, Manchester M13 9PL, U.K.

**Keywords:** ring current, aromaticity, density functional theory, porphyrin nanobelts, molecular electronics

## Abstract

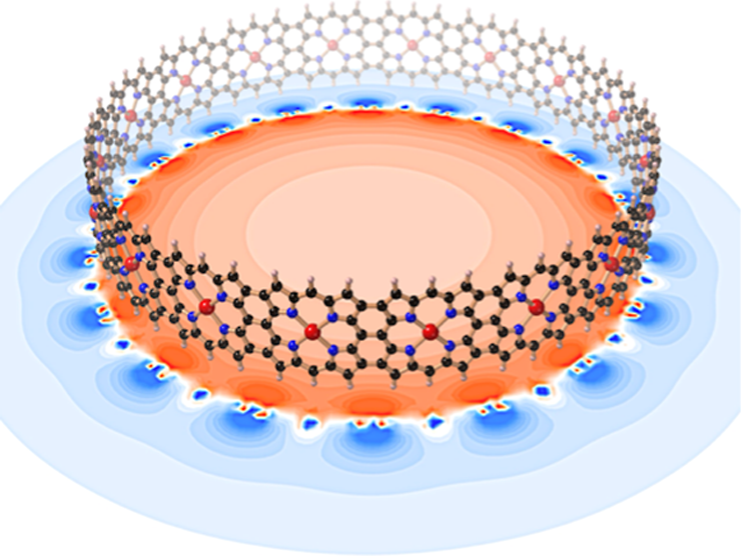

The ability of a ring-shaped molecule to sustain a global aromatic or antiaromatic ring current when placed in a magnetic field indicates that its electronic wave function is coherently delocalized around its whole circumference. Large molecules that display this behavior are attractive components for molecular electronic devices, but this phenomenon is rare in neutral molecules with circuits of more than 40 π-electrons. Here, we use theoretical methods to investigate how the global ring currents evolve with increasing ring size in cyclic molecular nanobelts built from edge-fused porphyrins. Our results indicate that a global ring current persists in neutral nanobelts with Hückel circuits of 220 π-electrons (22 porphyrin units, circumference 18.6 nm). Our predictions are validated by using coupled clusters to construct a density functional approximation (denoted as OX-B3LYP) that accurately describes these nanobelts and by checking compliance with Koopmans’ theorem.

## Introduction

**Figure 1:**
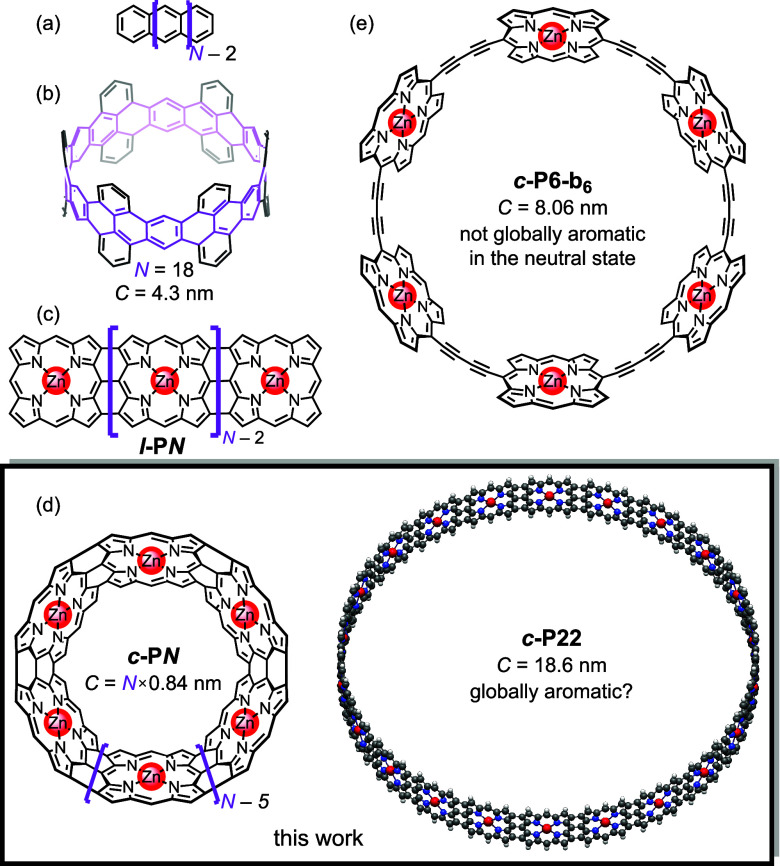
Conjugated systems for molecular electronics. (a) Acenes. (b) First reported zigzag-type carbon nanobelt (ref (3)), with the circumference (*C*) indicated. (c) Edge-fused nanoribbons ***l**-***P*****N***. (d) Edge-fused nanobelts ***c**-***P*****N*** investigated in this work. (e) 6-Porphyrin butadiyne-linked nanoring, ***c*****-P6-b**_**6**_.

The miniaturization of integrated circuits using top-down fabrication has reached a limit set by deleterious quantum effects, such as tunneling, which start to appear at the scale of a few nm.^1^ Bottom-up fabrication with molecular electronic components offers a fascinating alternative, as well-designed single-molecule devices can exploit quantum effects instead of being limited by them.^2^ Linear acenes and carbon nanobelts^3,4^ (Figure 1a,b) are attractive candidates for this purpose because they demonstrate efficient charge transport (*G* ≈ 0.1 *G*_0_, where *G*_0_ is the conductance quantum).^5^ Their porphyrin analogues, the edge-fused porphyrin nanoribbons ***l*****-P*****N*** (Figure 1c) show similarly high conductance (*G* ≈ 0.1 *G*_0_) but over longer distances,^6^ as well as exceptionally low energy gaps^7^ and quantum interference effects.^8^ These properties are attributed to extensive delocalization of the electronic wave function. In this work, we explore electronic delocalization in edge-fused porphyrin nanobelts^9^***c*****-P*****N*** (where *N* is the number of porphyrin units, Figure 1d) computationally, with the goal of guiding future synthetic exploration.

In molecular rings, a coherently delocalized electronic wave function causes the appearance of a global ring current under an externally applied magnetic field. This ring current produces magnetic shielding patterns that may be described using a particle-on-a-ring model. Hückel’s rule states that systems with circuits of 4*n* + 2 π-electrons (where *n* is an integer) sustain a diatropic current associated with aromaticity, while 4*n* π-systems sustain an antiaromatic paratropic current.^10,11^ As ring size increases, ring currents tend to become weaker due to symmetry-breaking and structural flexibility, which interrupt electronic delocalization.^12^

NMR spectroscopy has revealed substantial global ring currents in butadiyne- (Figure 1e) and ethyne-linked porphyrin nanorings with circuits of up to 162 π-electrons (*n* = 40).^12−14^ These singly linked nanorings only show global ring currents in their charged states because charging disrupts local aromaticity, and Coulombic repulsion promotes a more uniform charge distribution. In contrast, here we show that large triply linked ***c*****-P*****N*** nanobelts (Figure 1d) are expected to exhibit global ring currents even in the neutral state, due to the stronger coupling between the porphyrin subunits. This work is part of a wider experimental and computational project exploring the frontier between molecular rings and top-down fabricated nonmolecular nanorings that also exhibit persistent ring currents.^12,15,16^

While the experimental identification of global ring currents using NMR spectroscopy is often unambiguous, theoretical modeling is more challenging, particularly when using density functional theory (DFT) and nucleus-independent chemical shift (NICS) calculations.^17^ This is not surprising, as the extent of symmetry breaking and electronic delocalization in conjugated systems is sensitive to the choice of method,^18−21^ as first noted by Longuet-Higgins and Salem in 1959.^22^ This is especially apparent in the case of hybrid DFT, which builds on pure Kohn–Sham DFT by including a proportion of exact exchange (EE) in the density functional. Adding EE reduces the self-interaction error, which is one of the main weaknesses of pure DFT, but adding too much EE causes too much localization by underestimating dynamic correlation, which pure DFT generally handles very well.^23^

**Figure 2:**
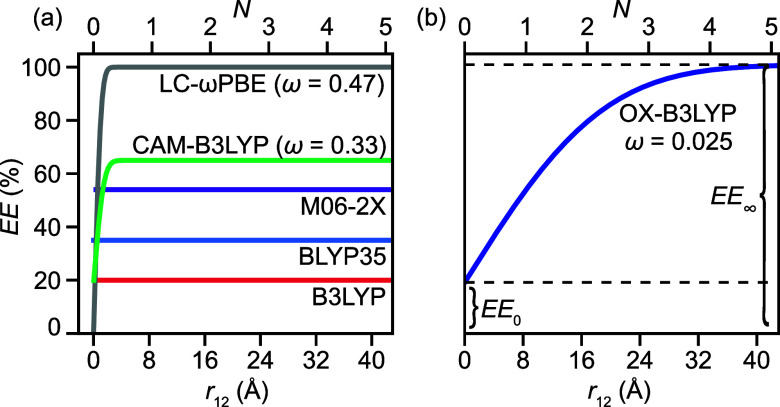
Variation of EE with interelectronic distance (*r*_12_) in (a) popular hybrid DFAs and (b) OX-B3LYP, with ω values (in units of *a*_0_^–1^) shown. *N* in the upper length scales indicates the number of edge-fused porphyrin units.

The difficulty of striking the right balance between localization (too much EE) and delocalization (too little EE) can be illustrated by the case of the butadiyne-linked 6-porphyrin nanoring in its 6+ oxidation state (***c**-***P6-b**_**6**_^**6+**^, Figure 1e). For this system, B3LYP^24^ (20% EE) overestimates the experimentally measured global ring current,^14,16^ M06-2X^25^ (54%) underestimates it,^26^ while BLYP35 (35%) reproduces the experimental results.^27^ M06-2X was also used to study magnetic properties of the antiaromatic ***c**-***P6-b**_**6**_^**4+**^ nanoribbons and of free-base ***l*****-P***N* arrays,^28^ while B3LYP was used for the ***c**-***P40** nanoring.^29^ All of these DFAs have a constant proportion of EE (red, light blue, and purple in Figure 2a), which results in an incorrect description of the Coulomb interaction in the long-range limit and a poor description of charge transfer. Range-separated DFAs (e.g., LC-ωPBE,^30^ gray in Figure 2a), in which the proportion of EE is dependent on the interelectronic distance *r*_12_, remedy this issue^31,32^ but need three tunable parameters: EE_0_ and EE_∞_, which control the proportion of EE at the short- (*r*_12_ = 0) and long-range (*r*_12_ = ∞) limits, and ω (usually given in units of reciprocal Bohr radius, *a*_0_^–1^, with *a*_0_ ≈ 0.53 Å), which controls the transition from EE_0_ to EE_∞_ (Figure 2b). In the case of the above-mentioned ***c**-***P6-b**_**6**_^**6+**^, the range-separated functionals CAM-B3LYP^33^ (green in Figure 2a) and a modified flavor of LC-ωPBE (ω = 0.1 *a*_0_^–1^)^30^ were both used to model its ring current, with good results.^26,27,34^

At this point, the skeptical reader may rightly ask: if choosing a suitable DFA for modeling ring currents in nanorings for which experimental data *are* available is so difficult, what hope do we have of accurately predicting ring currents in ***c*****-P*****N*** nanobelts, which have yet to be synthesized? To answer this question, we recall that the equilibrium geometry of a conjugated system will be the result of a balance between the (distortive) π- and the (restorative) σ-electrons.^20,35,36^ This balance will be highly dependent on the proportion of EE in hybrid DFT. Therefore, if we build a series of DFAs with different proportions of EE, the DFA providing the most accurate equilibrium geometry is likely to have the correct balance and can be expected to give a reliable description of the electronic structure and ring current. We have recently shown that this hypothesis holds well in case of [18]annulene, an archetypal conjugated system.^37^ Using coupled clusters energies as reference (which are free from self-interaction error, but also include dynamic correlation), we identified BLYP45 (45% EE) as the optimal DFA and found that it reproduces chemical shifts in both [18]annulene and its anions with a <1 ppm accuracy over a >30 ppm range. Similar approaches have been used successfully to identify appropriate DFAs for modeling interconversions between Hückel and Möbius topologies,^38^ fullerene-based memristors,^39^ and pericyclic reactions.^40^

## Results and Discussion

In this work, we adopted the following strategy for identifying a DFA suitable for modeling ***c**-***P*****N*** nanobelts (details in Supporting Information Section A):

1 Optimize geometries of various ***c**-***P*****N*** systems using several families of DFAs (PBE,^41^ BLYP,^24^ and ωB97X^42^). Within each family, test many DFAs by changing the proportion of EE or EE_0_, EE_∞_, and ω.
2 Calculate the single-point energy for each optimized geometry of each ***c*****-P*****N*** using coupled clusters (or second-order perturbation theory, MP2, for larger belts). Identify the DFA that minimizes this energy for each value of *N.*
3 Keeping in mind that coupled clusters and MP2 calculations are sensitive to basis size,^43^ refine the DFA by correcting for basis set incompleteness.

Following the strategy outlined above, we built OX-B3LYP (Optimized for eXtensively conjugated systems, dark blue in Figure 2b) as the optimal DFA for describing the ***c**-***P*****N*** nanobelts. At small *r*_12_, OX-B3LYP (EE_0_ = 19%) is almost identical to B3LYP (EE = 20%), which is a suitable DFA for modeling the optical properties of edge-fused porphyrin nanoribbons ***l*****-P*N*** (Figure 1a; see Supporting Information Section B).^44^ In the long-range limit, OX-B3LYP recovers the correct form of the Coulombic decay (EE_∞_ = 100%).

The most interesting feature of OX-B3LYP is the very low value of the range separation parameter (ω = 0.025 *a*_0_^–1^). This can be understood by noting that 1/ω roughly corresponds to the distance at which electron–electron interactions are no longer screened (when EE_∞_ = 100%). In general-purpose range-separated DFAs such as the original LC-ωPBE (ω = 0.47 *a*_0_^–1^, gray in Figure 2a), 1/ω is comparable to the length of a single bond (1/ω = 1.1 Å). In the LC-ωPBE variant suitable for singly linked porphyrins (ω = 0.1 *a*_0_^–1^),^14,26,27,34,45^ 1/ω is on the order of a single porphyrin (5.3 Å), indicating that electrons are strongly delocalized within each porphyrin, but that inter-porphyrin interactions are unscreened. In OX-B3LYP (dark blue in Figure 2b), screening persists up to several porphyrins in length (1/ω = 21.2 Å), reflecting strong inter-porphyrin coupling in ***c*****-P*****N*** nanobelts.

An alternative nonempirical approach for tuning a DFA for a specific system relies on making it compliant with Koopmans’ theorem, which is usually accomplished by varying ω so that the negative HOMO energy matches the vertical electron detachment energy of the neutral molecule.^46−48^ Applying this procedure to ***c**-***P6**, we obtain ω = 0.030 *a*_0_^–1^ as the optimal value, which is very close to ω = 0.025 *a*_0_^–1^ in OX-B3LYP (see Supporting Information Section c). Therefore, two unrelated nonempirical tuning procedures, based on the minimization of coupled cluster energy or compliance to Koopmans’ theorem, independently produce very similar estimates for the limit of global aromaticity. Finally, thermal movement does not appear to dampen the ring current (see Supporting Information Section D).

**Figure 3:**
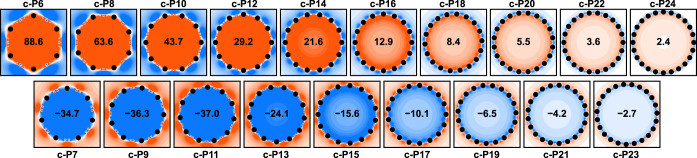
NICS(0)_*zz*_ maps for ***c**-***P*****N*** nanobelts with *N* = 6–24, computed using OX-B3LYP. The NICS(0)_*zz*_ value in the center of the ring is shown in bold.

We used OX-B3LYP to evaluate the ring currents and aromaticity in ***c*****-P*****N*** nanobelts. Each porphyrin unit in a ***c*****-P*****N*** nanobelt contributes 10 electrons to the Hückel count for the global ring current (see Figure S16),^16,49^ so we expect even-numbered belts to sustain a paratropic ring current associated with antiaromaticity (positive NICS inside the ring; top row in Figure 3) and the odd-numbered ones to display a diatropic current consistent with aromaticity (negative NICS inside the ring; bottom row in Figure 3). Using OX-B3LYP, we find that this is indeed the case and predict ***c**-***P22** (circumference of 18.6 nm) as the largest nanobelt to show a ring-current-induced shift at the center of the ring larger than 1 ppm (NICS(0)_*zz*_ of ±3 ppm corresponds to an isotropic shift of ±1 ppm, which is roughly the accuracy of DFT;^50,51^Figure 3). We also note that the best-performing DFA with a constant proportion of EE (BLYP25) provides a very similar estimate (Figure S5).

**Figure 4:**
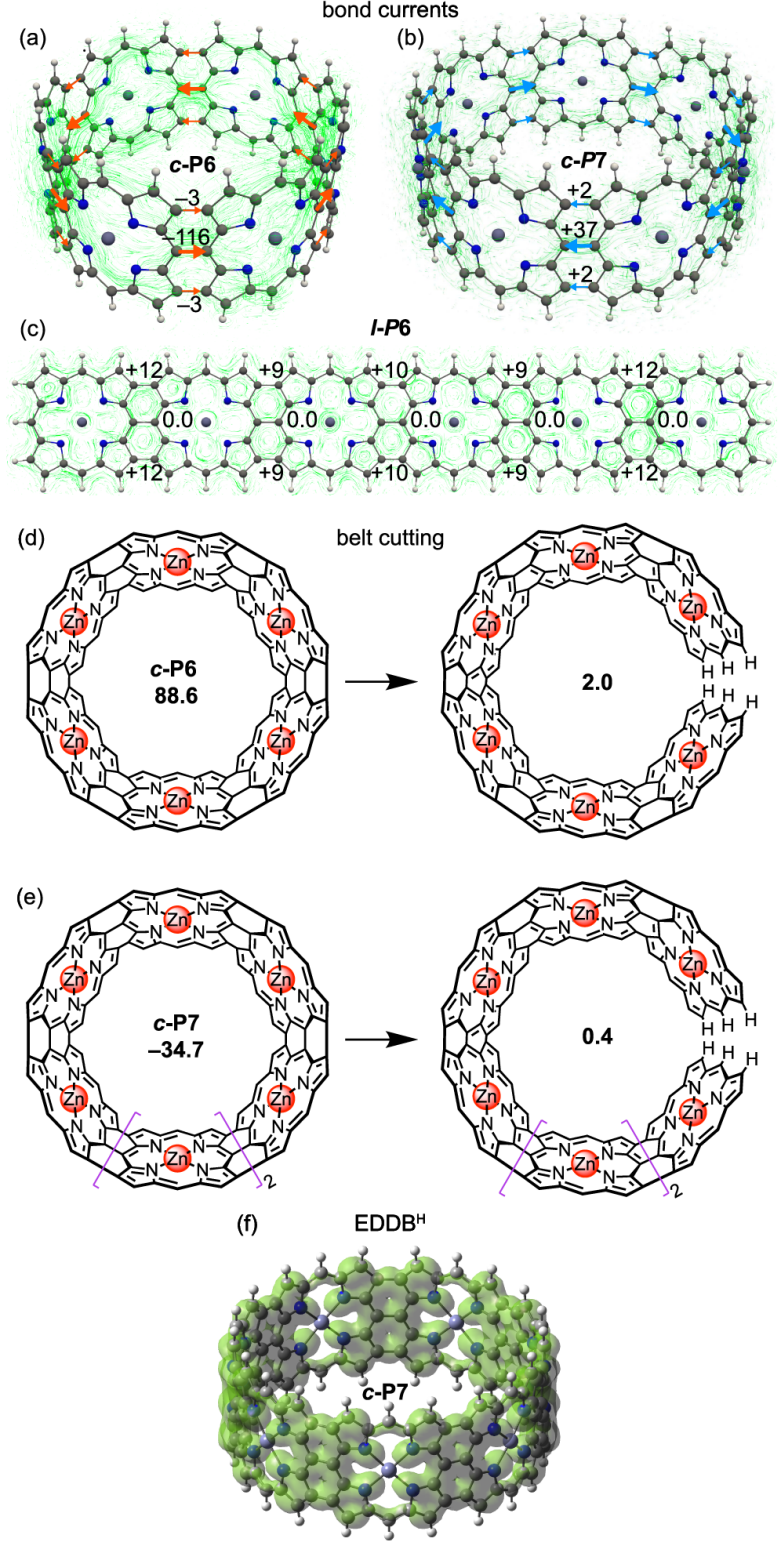
(a–c) Induced currents (green lines) and bond current strengths (in nA/T) in the bonds linking the individual porphyrins in (a) ***c*****-P6**, (b) ***c*****-P7**, and (c) ***l*****-P6**, induced by a magnetic field perpendicular to the belt. Paratropic (diatropic) bond currents are shown in red, counterclockwise, and negative (blue, clockwise, and positive). (d,e) Change in NICS(0)_*zz*_ upon belt opening (d) ***c*****-P6** and (e) ***c*****-P7**. (f) EDDB_H_ in ***c*****-P7** plotted at an isovalue of 0.02 au.

Although NICS is useful, it does not always definitely prove the presence of a global ring current.^52^ To do so, we visualized the induced currents using the GIMIC (gauge-including magnetically induced currents) approach^53,54^ in ***c*****-P6**, ***c*****-P7**, and ***l*****-P6** (Figure 4a–c and Supporting Videos 1 and 2). The belts (Figure 4a,b) show global circulation which switches direction between *N* = 6 and *N* = 7, demonstrating the presence of a global ring current; in contrast, global circulation is absent in the linear nanoribbon (Figure 4c; previous work suggested a weak diatropic current^28^). GIMIC calculations also revealed that the individual porphyrins retain their local aromaticity regardless of the direction of the global current, as shown previously in similar nanorings.^55^

The suitability of using NICS(0)_*zz*_ at the center of the belt as a probe of the global current can be demonstrated by creating a break in the belt while keeping it roughly the same shape (Figure 4d,e). This disrupts the global ring current but does not prevent the local currents from affecting the NICS probe. We found that cutting the belt decreases NICS(0)_*zz*_ at the belt center to nearly zero, confirming that the calculated values in Figure 3 can be attributed to the global current.

The presence of global currents was also confirmed by calculating the bond currents^56^ in the bonds linking the porphyrins (Figure 4a,b). Orbital-decomposed analysis of these bond currents shows that the paratropic component of the global current in ***c*****-P6** (∼80% of the total current) can be accounted for by just two electrons, while four electrons are responsible for ∼90% of the diatropic current in ***c*****-P7** (Figure S13).

We also quantified the electron delocalization in these nanobelts using EDDB (electron density of delocalized bonds)^57^ (Figure 4f). EDDB^H^ values indicate that all investigated nanobelts have more delocalized electrons (∼25*N*) than their ribbon analogues (24.0–24.4*N*), but they do not conclusively show the size limit of the global ring current (Figure S11).

Changing the metal in a metalloporphyrin can be a useful way to tune its electronic structure. Nickel porphyrins have similar aromaticity as their zinc analogues, but due to their vacant *d*_*x*^2^–*y*^2^_ orbitals, they tend to have larger HOMO–LUMO gaps and lower HOMO levels, resulting in blue-shifted absorption spectra and lower chemical reactivity.^58^ Using OX-B3LYP, we have calculated NICS(0)_*zz*_ for ***c-***^**Ni**^**P*****N*** with even *N* = 8–20, revealing that the 14-porphyrin ring is the largest nickel porphyrin-based nanobelt to display a global ring current, in contrast to the zinc complexes in which global ring currents persist up to *N* = 22 (see Supporting Information Section E). A related effect is observed in linear ***l*****-P*****N*** ribbons, in that infinite nickel metalated ribbons are calculated to have a wider band gap than the corresponding zinc ribbons.^58^

**Figure 5:**
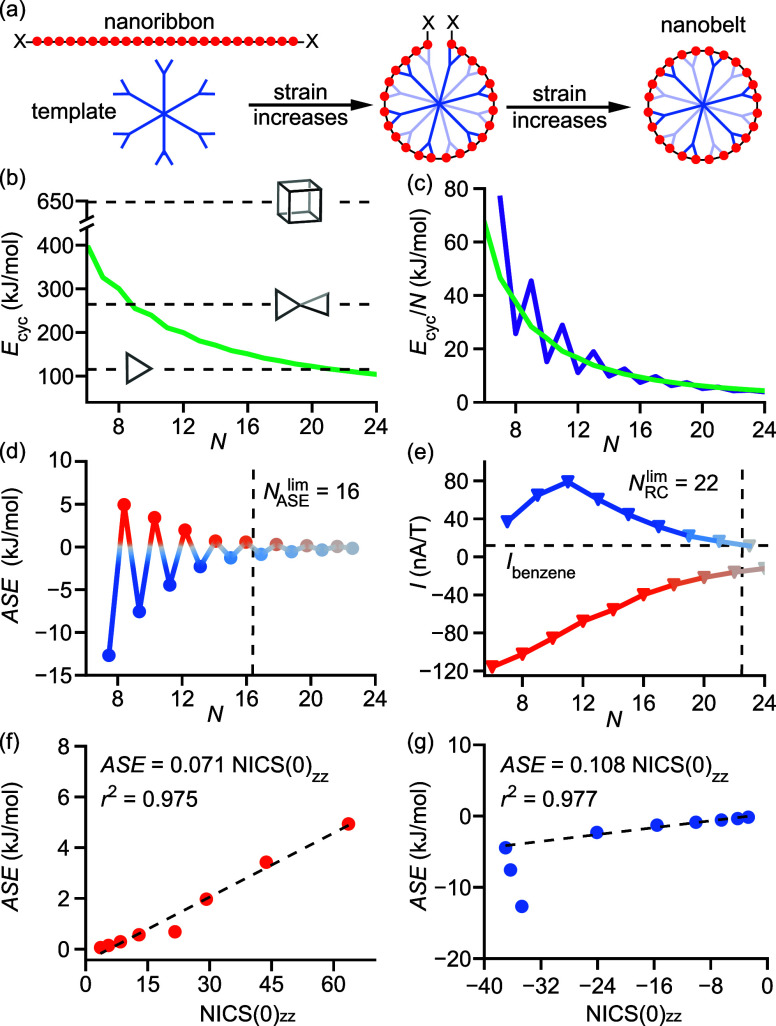
(a) Template-directed porphyrin nanoring synthesis. (b) Cyclization energy *E*_cyc_ (in kJ/mol) in ***c**-***P*****N*** nanobelts, compared to archetypal strained systems, cubane, bicyclopentane, and cyclopropane (indicated by horizontal dashed lines). (c) Cyclization energy per porphyrin (green) and energy release associated with increasing the belt size from *N* to *N* + 1 (purple). (d) ASE per porphyrin, with the limiting value with |ASE| > 1 kJ/mol denoted. (e) Integrated bond current, with the value for benzene (12 nA/T) denoted. (f,g) Correlation between NICS(0)_*zz*_ and ASE for even- (f) and odd- (g) numbered belts, with linear fit parameters. *r*^2^ denotes the coefficient of determination, and the intercept is set to zero. The anomalously small ring currents in belts with *N* = 7, 9 (in e,g) are due to a combination of high strain and low symmetry (*C*_2v_ vs *D*_*N*h_ in even-*N* belts) and are excluded from the linear fit.

We now shift our attention to evaluating the possibility of making these nanobelts. Template-directed synthesis is often used to prepare porphyrin nanorings,^59−61^ starting conceptually from a porphyrin nanoribbon and then bending it into a ring (Figure 5a). This cyclization is associated with a considerable increase in energy due to strain. Here, we consider the energy difference between linear and cyclic ribbons, the cyclization energy *E*_cyc_, which is made up of the strain energy, together with the aromatic (de)stabilization energy (ASE) associated with the cyclization. We used OX-B3LYP and a hyperhomodesmotic^62^ scheme (details in Supporting Information Section F) to show that the cyclization energy *E*_cyc_ in ***c*****-P*****N*** nanobelts decreases rapidly until about *N* > 12, then declines more gradually (Figure 5b,c) with increasing *N*. Combining these values with our NICS calculations, we can identify *N* = 16–18 nanobelts as the most attractive candidates for synthesis, offering relatively low strain and significant global ring currents. The belts for *N* = 6–18 are less strained than cubane but more strained than cyclopropane, although the strain is spread over a large number of bonds.^63^

Aromatic stabilization energy (ASE) is a popular measure of aromaticity, calculated as the difference in energy between an (anti)aromatic cyclic system and a nonaromatic analogue, which may be linear or cross-conjugated.^64^ Classic examples are cyclobutadiene and benzene, which have higher and lower energy, respectively, than their nonaromatic counterparts, corresponding to positive and negative ASE. OX-B3LYP calculations of ***c*****-P*****N*** nanobelts reveal similar behavior, with even-*N* belts (with 4*n* π-electrons) displaying a positive and odd-*N* belts (with 4*n* + 2 π-electrons) a negative ASE (Figure 5d). Here, we calculate the ASE as the difference in energy between a particular ***c*****-P*****N*** and the smooth curve without the oscillation between aromatic and antiaromatic species, thus avoiding the common problem of defining a nonaromatic reference system (Supporting Information Section F).^45^ The reported ASE values are relative to ***l*****-P*****N***, i.e., they represent the additional (de)stabilization associated with the global ring current, and they are not very sensitive to the employed DFA (see Figure S10).

We find that the magnitude of ASE is very small, dropping from ∼13 kJ/mol at *N* = 6 to ∼1 kJ/mol at *N* = 16 (Figure 5d), which suggests that these belts are not (anti)aromatic according to the energetic criterion. On the other hand, magnetic criteria strongly favor global (anti)aromaticity, with NICS values (Figure 3) and bond current calculations (Figure 5e) showing global circulation up to *N* = 22, where the current is comparable to benzene (12 nA/T). These conflicting results may be resolved by recognizing that ASE and NICS values are both inversely proportional to the ring circumference, which can be shown by Hückel theory^45^ and the Biot-Savart law, respectively. While both ASE and NICS will unavoidably reach zero as the ring size is increased, they are highly correlated (Figure 5f,g), indicating that they describe the same phenomenon.

## Conclusions

Recognizing the importance of balancing the description of localization and delocalization in conjugated systems, here, we used coupled clusters as a reference to build OX-B3LYP, a DFA specifically tuned for the description of edge-fused porphyrin nanobelts. OX-B3LYP predicts the presence of global ring currents in neutral ***c*****-P*****N*** belts with up to 22 porphyrin units, indicating global (anti)aromaticity, according to the magnetic criterion, in molecular rings with a Hückel circuit of 220 π electrons and a circumference *C* = 18.6 nm. This finding considerably pushes the size limit of the global aromaticity. To our knowledge, the largest macrocycle displaying a global ring current in its neutral state reported until now had 54 π electrons (aromatic, *C* ≈ 5.0 nm).^65−67^***c*****-P*****N*** belts are appealing components for molecular electronics, and they might be particularly suitable for devices which benefit from a large ring circumference, such as Aharonov–Bohm interferometers.^68^

The size limit of aromaticity may be pushed even further if charged systems are considered. For example, the butadiyne-linked 12-porphyrin ring ***c**-***P12-b**_**12**_ (*C* = 16.1 nm) displays a ring current in the charged (e.g., 6+) states but not in the neutral molecule.^14^ Indeed, an OX-B3LYP calculation for ***c*****-P24**^**12+**^ (*C* = 20.3 nm) yields NICS(0)_*zz*_ = 25.6 ppm (using a geometry optimized at GFN2-xTB^69^), indicating a 10-fold increase of the ring current upon charging.

## Methods

All geometry optimizations and NICS calculations employed the def2-SVP basis set and were done using Gaussian16.^70^ Local CCSD(T) and CCSD(T)-F12^43^ single-point energies were evaluated using ORCA,^71^ while spin component scaled MP2^72^ energy evaluations were done using Turbomole.^73^ Bond currents were calculated using SYSMOIC,^56^ while induced currents were determined from DFT density using GIMIC.^53,54^

## Supporting Information Available

Input cards for OX-B3LYP, results for DFA tuning using coupled clusters, performance of various DFAs in modeling ***l*****-P*****N*** absorption spectra, Koopmans’ theorem-based tuning, effects of thermal motion on NICS, NICS of nickel porphyrins, cyclization and strain analysis, other supplementary figures, and additional input files (PDF)
Animated representations of the global ring current in ***c*****-P6** (MOV)
Animated representations of the global ring current in ***c*****-P7** (MOV)

## Supplementary Material

nn4c14100_si_001.pdf

nn4c14100_si_002.mov

nn4c14100_si_003.mov

## Notes

**Preprint**: A previous version of this manuscript was deposited on the ChemRxiv preprint server: Vitek, M.; Deng, J.-R.; Anderson, H. L.; Rončević, I. Global Aromaticity in Neutral Porphyrin Nanobelts. **2024**. *ChemRxiv*. DOI: 10.26434/chemrxiv-2024-vj96d-v3 (accessed Dec 09, 2024).
