# Supplementary material for: Global Aromatic Ring Currents in Neutral Porphyrin Nanobelts
Source: ACS Nano. 2024 Dec 31;19(1):1405–11. doi: 10.1021/acsnano.4c14100 (PMC11752501; doi:10.1021/acsnano.4c14100)
Supplement: Supplementary file 1 — nn4c14100_si_001.pdf [file nn4c14100_si_001.pdf]

Global Aromaticity in Neutral Porphyrin Nanobelts

Marco Vitek,[a,b] Jie-Ren Deng,[a] Harry L. Anderson,[a] Igor Rončević*[a,c]

a Marco Vitek, Jie-Ren Deng, Harry L. Anderson, Igor Rončević
Department of Chemistry, University of Oxford, Chemistry Research Laboratory, Oxford OX1 3TA, United Kingdom
b Marco Vitek
Institute of Organic Chemistry and Biochemistry of the Czech Academy of Sciences
Flemingovo nám. 542/2, 160 00 Prague 6, Czechia
c Igor Rončević
Department of Chemistry, The University of Manchester, Oxford Road, Manchester M13 9PL, United Kingdom
E-mail: igor.roncevic@manchester.ac.uk

Input cards for OX-B3LYP *1*
(A) Tuning the DFA using coupled clusters energies as reference. *2*
(B) Performance of DFAs in modelling edge-fused nanoribbon absorption spectra *4*
(C) Koopmans' theorem-based tuning. *5*
(D) Temperature dependence of aromaticity. *5*
(E) Nickel porphyrins. *6*
(F) Cyclization, strain, and ASE. *6*
(G) Other supplementary figures. *7*
(H) Representative input files. *9*
References: *13*

All optimized geometries are available as a Zenodo dataset (DOI: 10.5281/zenodo.12744769).

Input cards for OX-B3LYP

Gaussian16:

```
# Single point energy calculation with OX-B3LYP (w=0.025, EXHF: 19% => 100%) in Gaussian16
%nproc=36
%mem=72GB
#p def2svp cam-b3lyp
IOp(3/107=0025000000,3/108=0025000000,3/119=0810000000,3/120=0810000000,3/130=01900,3/131=01900)
```

ORCA:

```
# Single point energy calculation with OX-B3LYP (w=0.025, EXHF: 19% => 100%) in Orca
! CAM-B3LYP def2-SVP def2-SVP/C def2/J RIJCOSX TightSCF

%method
    RangeSepEXX True
    RangeSepMu 0.025
    RangeSepScal 0.81 # should sum to 1 with ACM-A and ACM-B
    ACM 0.19, 0.00, 0.81 # ACM-A, ACM-B, ACM-C
end
```

Turbomole:

```
$dft
   functional cam-b3lyp_own 0.19 0.81 0.025
   gridsize   m5
```

(A) Tuning the DFA using coupled clusters energies as reference.
This procedure was done in four steps:

1. Optimization of ***c*-P*N*** (*N* = 6–24) geometries using DFA/def2SVP,[1] where DFA stands for a series of 'flat' (with a constant proportion of exact exchange, EE) or range-separated density functional approximations based on the PBE, BLYP, and ωB97X families. Dispersion corrections were omitted, as they are not available for custom-made DFAs. Solvent effects were not included. These calculations were done using Gaussian16.[2] Representative input files are given in Section H1.

2. To evaluate the performance of different DFAs, single-point energies of geometries optimized in (1) were evaluated at the DLPNO-CCSD(T)/cc-pVTZ level of theory[3] for rings with *N* = 6,7 (Figure S1). For larger rings such calculations were not feasible, so SCS-MP2[4] was used for *N* = 6–14 (Figure S2). SCS-MP2 was chosen as the best candidate by screening the performance of several double hybrid DFAs and flavours of second-order perturbation theory (Figure S3).These calculations utilized ORCA[5] and Turbomole[6]. Representative input files are given in H2.

3. Results from (1) and (2) identified BLYP30 and a variant of OX-B3LYP (EE ranging from 19–100%, $\omega = 0.1\ a_0^{-1}$) as the best-performing DFAs. However, coupled clusters energies is well-known to converge slowly with basis set size.[7] To correct for basis set completeness, we evaluated the performance of BLYPxx (varying EE) and OX-B3LYP (varying *ω*) against DLPNO-CCSD(T)-F12 single point energies on a zinc porphyrin monomer. These calculations were done using ORCA.[5] Our results (Figure S4) indicated that lower proportions of EE (25% in the case of BLYP and $\omega = 0.025\ a_0^{-1}$ in the case of OX-B3LYP) are needed. This is not a surprising result, as increasing the basis set size in coupled clusters methods results in a more complete account of dynamic correlation (as more excited configurations are taken into account). More dynamic correlation increases delocalization (by screening the Columbic potential), which corresponds to lowering the proportion of EE in DFT-based methods. Representative input files for OX-B3LYP are provided above, and the remainder are given in Section H3.

4. Finally, NICS(0)$_{zz}$ values were calculated using OX-B3LYP ($\omega = 0.025\ a_0^{-1}$) and BLYP25 at the equilibrium geometry of those respective DFAs. Results for OX-BL3YP are shown in Figure 3 (main text) and for BLYP25 in Figure S5. In the main text we gave preference to OX-B3LYP because (a) it provides a better account of absorption spectra of edge-fused porphyrin tapes (Figure 1a; also see section B), and (b) the Koopmans' theorem-based tuning procedure (see section C) suggests that it is suitable for modelling ***c*-P6**. We note that both functionals estimate the size limit of global aromaticity around *N* = 20. Representative input files are given in H4.

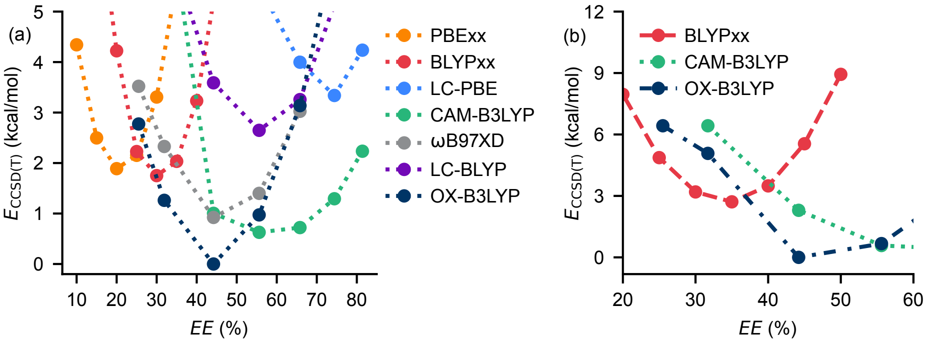

Figure S1. Comparison of single-point CCSD(T) energies of geometries optimized using various DFAs for ***c*-P6** (a) and ***c*-P7** (b). DFAs appended with xx have a flat proportion of exact exchange (EE). In range-separated DFAs we varied *ω*, and the proportion of EE at 1.5 Å (typical C-C bond length) is plotted.

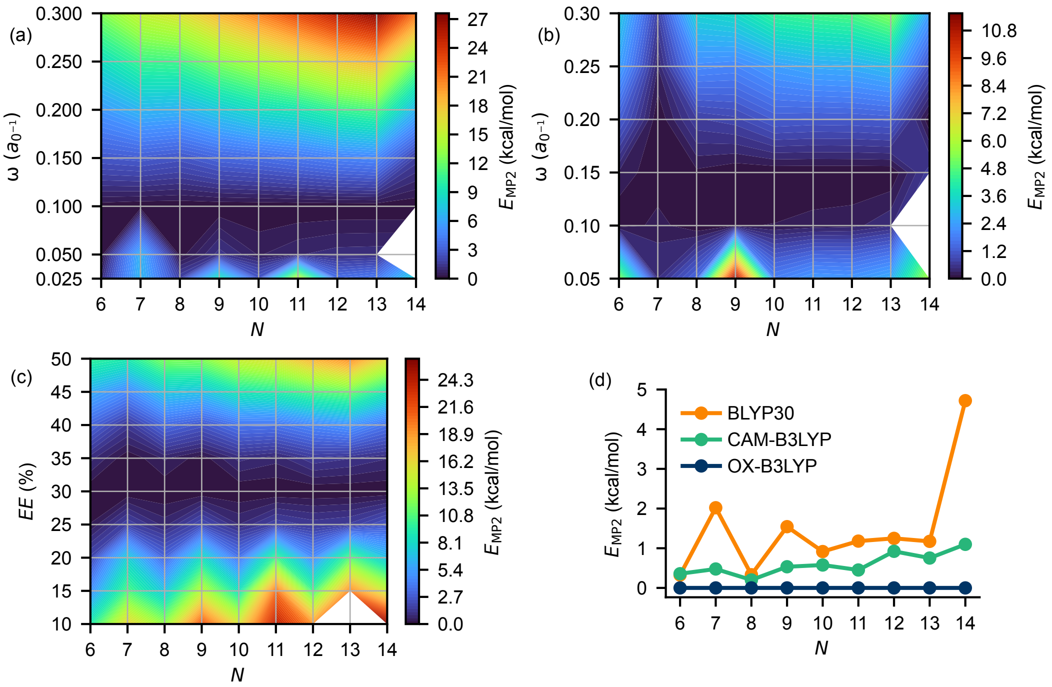

Figure S2. MP2 energies for ***c*-P*N*** (*N* = 6–14). (a) Heatmap of MP2 energies for different EE percentages in BLYPxx as a function of *N*. (b) Heatmap of MP2 energies for different *ω* values in CAM-B3LYP as a function of *N*. (c) Heatmap of MP2 energies for different ω values in OX-B3LYP as a function of *N*. (d) Plot of the lowest MP2 energy for each functional (BLYP30, CAM-B3LYP, and OX-B3LYP) as a function of *N*.

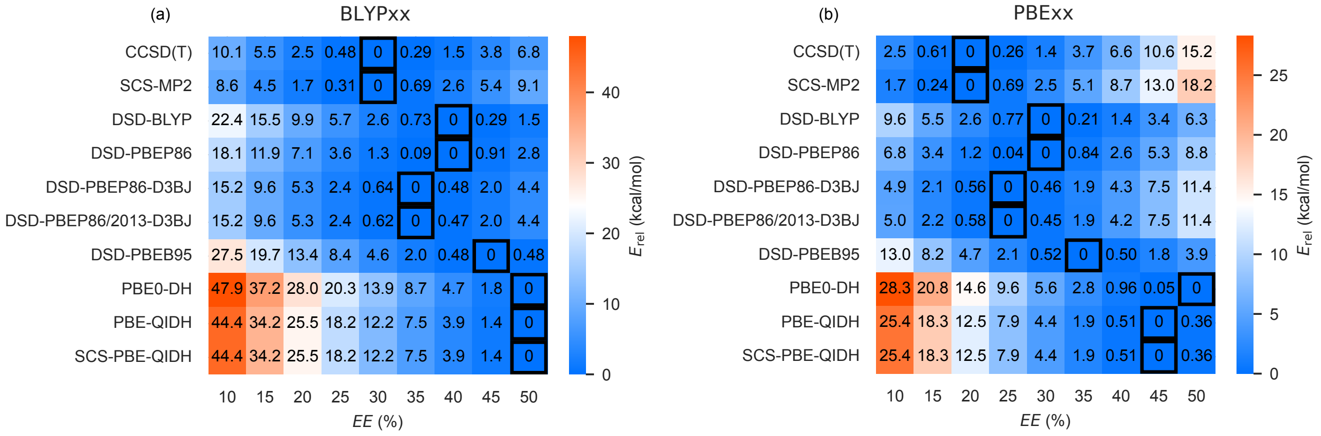

Figure S3. Performance of various DFAs and MP2 in relative to CCSD(T) for ***c*-P6**. (a) Relative energies of geometries optimized using BLYPxx with varying proportions of EE compared to CCSD(T) reference energies, including results from MP2 and several double hybrid DFAs. (b) Relative energies of geometries optimized using PBExx with varying proportions of EE compared to CCSD(T) reference energies, including results from MP2 and several double hybrid DFAs. In both cases (a,b), MP2 closely matches the CCSD(T) results, identifying the same EE configuration with the lowest energy.

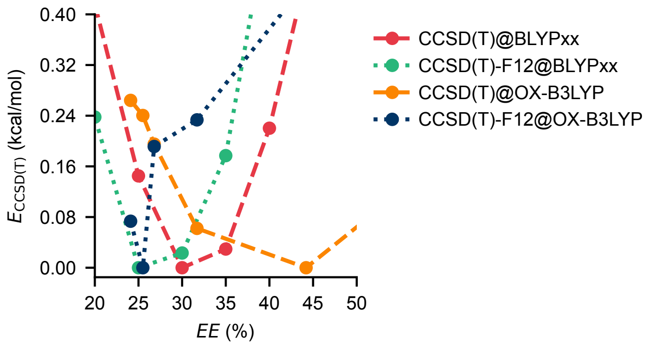

Figure S4. Performance evaluation of BLYPxx and OX-B3LYP functionals with varying proportions of EE in comparison to CCSD(T) and CCSD(T)-F12 single-point energies for a zinc porphyrin monomer. In range-separated DFAs we varied *ω*, and the proportion of EE at 1.5 Å (typical C-C bond length) is plotted.

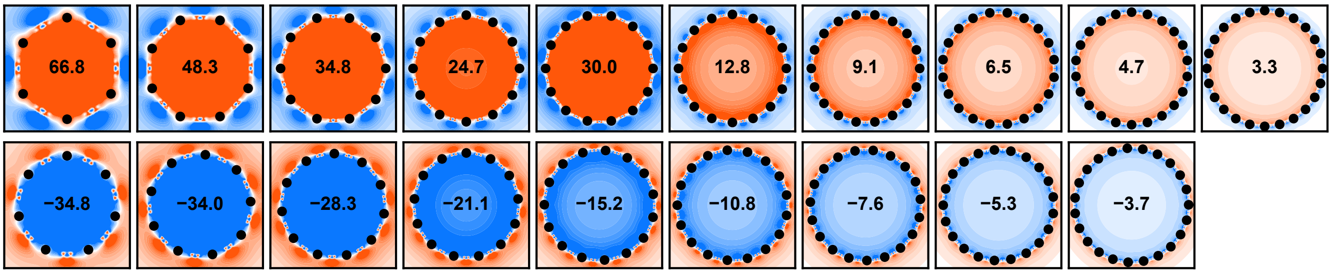

Figure S5. NICS(0)$_{zz}$ maps for ***c*-P*N*** nanobelts with N = 6–24, computed using BLYP25. The NICS(0)$_{zz}$ value in the center of the ring is shown in bold.

(B) Performance of DFAs in modelling edge-fused nanoribbon absorption spectra.

As the magnetic response is derived from the coupling between the occupied and unoccupied orbitals,[9,10] it is reasonable to expect that DFAs which give a good account of optical spectra will also accurately estimate the NICS values. The ***c*-P*N*** nanobelts have not been synthesized, but the absorption spectra of their tape analogues ***l*-P*N*** are well-known.[11] To further investigate the performance of different DFAs, we compared their predicted first vertical transition energies to experimental absorption maxima. Our results indicate that OX-B3LYP gives a very good agreement with experiment (Figure S6a), with a mean unsigned error of 0.07 eV, without including solvent effect or side chains. Comparable performance is obtained by B3LYP (green in Figure S6b), which was previously found to be an excellent choice for ***l*-P*N*** systems.[12] Representative input files are given in section H5.

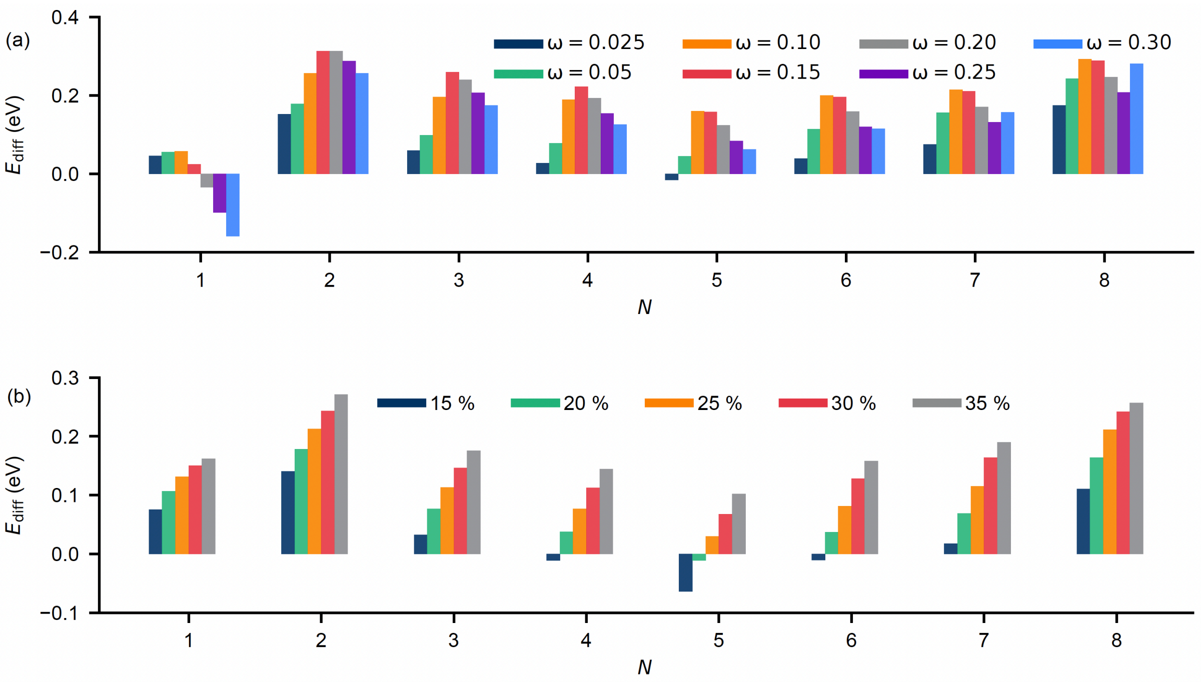

Figure S6. Performance of DFAs in modeling absorption spectra of edge-fused nanoribbons ***l*-P*N*** (N = 1–8). (a) Predicted first vertical transition energies for different ω values in OX-B3LYP as a function of N. (b) Predicted first vertical transition energies for different EE percentages in BLYPxx as a function of N. These results compare the DFAs' predicted first vertical transition energies to experimental absorption maxima.

(C) Koopmans' theorem-based tuning.
To determine the optimal ω value for the OX-B3LYP (EE from 19–100%) DFA, we adopted the following tuning procedure: for each value of ω, we optimized geometry and calculated $J^2 = (-E_H - E_{VED})^2$, where E_H is the HOMO energy and E_{VED} the vertical electron detachment energy, calculated as the energy difference from the radical cation and the neutral molecule. For ***c*-P6**, our tuning procedure indicates that an $\omega = 0.03\ a_0^{-1}$ is ideal, with values between 0.01 and 0.05 a_0^{-1} showing very good performance (Figure S7 a). This is in excellent agreement with the $\omega = 0.025\ a_0^{-1}$ obtained using the completely independent coupled clusters-based procedure in section (A), and the evaluation based on absorption spectra in section (B). We note that the ω value of 0.03 a_0^{-1} predicts virtually the same size limit of global aromaticity as 0.025 a_0^{-1} (Figure S7b,c).

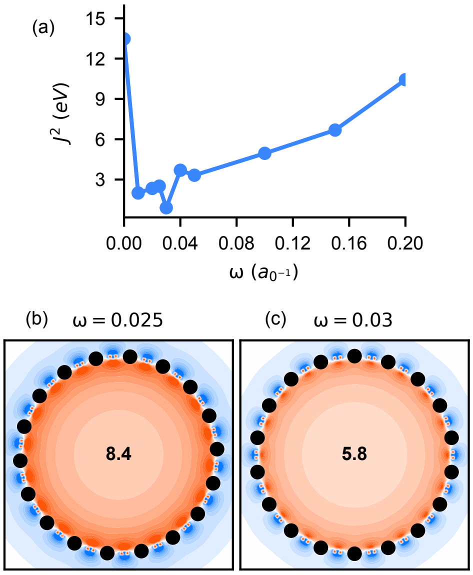

Figure S7. Koopmans' theorem-based tuning for determining the optimal ω value for the OX-B3LYP. Plot showing the relationship between ω values and J^2 for ***c*-P6** (a). (b,c) $NICS(0)_{zz}$ maps for ***c*-P18** with OX-BL3YP (b) and a variant of OX-BL3YP (ω = 0.03 a_0^{-1}) (c).

(D) Temperature dependence of aromaticity.
To assess how the NICS values change with thermal motion, we performed an MD simulation of ***c*-P18** at the semiempirical GFN2-xTB level of theory using the xTB code.[13] We used a total simulation time of 10 ps and the NVT ensemble with $T = 298.15$ K. Hydrogen masses were scaled by a factor of 4, which enabled us to use a 2 fs time step. To simulate the effect of the template, we constrained the Zn atoms with a harmonic potential of 0.0533 a.u., which effectively prevented them from moving more than 0.1 Å from their equilibrium positions.

Following the simulations, $NICS(0)_{zz}$ values were determined at the center of the ring in 20 snapshots taken at 0.5 ps interval using OX-B3LYP (Figure S8). These results showed significant fluctuations in $NICS(0)_{zz}$, suggesting that the global aromaticity is sensitive to thermal motions. However, the thermal movement does not appear to break the global aromaticity, and somewhat surprisingly, the $NICS(0)_{zz}$ values increase in most snapshots. Representative input files are given in section H6.

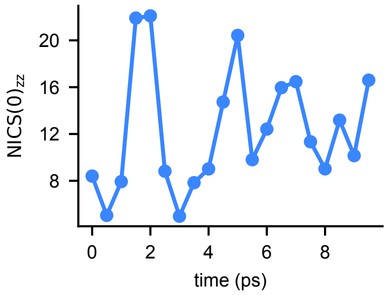

Figure S8. $NICS(0)_{zz}$ values over time for ***c*-P18** during a 10 ps MD simulation at 298.15 K, showing fluctuations in global aromaticity due to thermal motions.

(E) Nickel porphyrins.
Porphyrin-based systems with nickel instead of zinc are attractive due to their higher stability under harsh conditions. Such systems also tend to have larger HOMO-LUMO gaps due to a lower-lying HOMO, which may be due to a vacant d_{x2-y2} orbital on Ni.[14] OX-B3LYP calculations on ***c*-NiP*N*** suggest that global aromaticity vanishes at around $N = 18$ in these systems, which is unsurprising as their larger HOMO-LUMO gaps result in weaker magnetic coupling between the occupied and unoccupied orbitals.

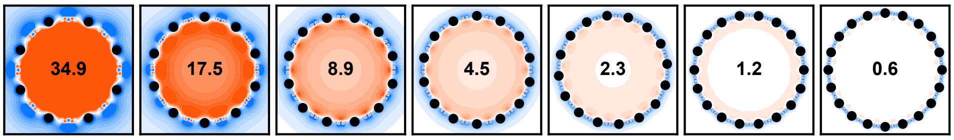

Figure S9. NICS(0)$_{zz}$ maps for ***c*-NiP*N*** (N = 8,10,12,14,16,18,20) nanobelts computed using OX-B3LYP. The NICS(0)$_{zz}$ value in the center of the ring is shown in bold.

(F) Cyclization, strain, and ASE.
Cyclization. The cyclization energy E_{cyc} of ***c*-P6** was obtained by evaluating the reaction energy of hyperhomodesmotic reaction (S1), while E_{cyc} of all other ***c*-P*N*** rings was determined by from (S2).

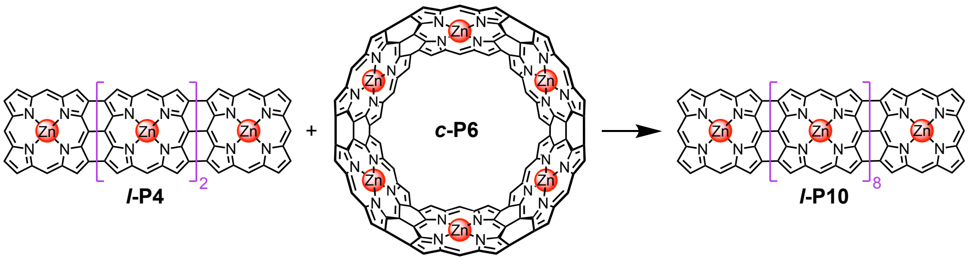

(S1)

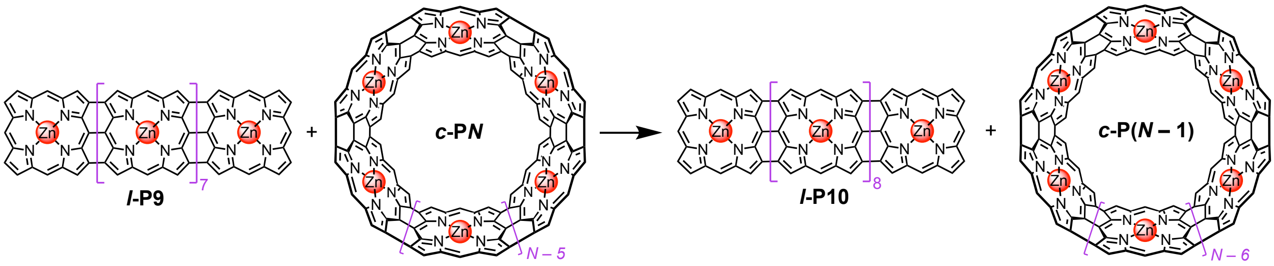

(S2)

Strain and ASE. The cyclization energy E_{cyc} can be written as a sum of the strain energy E_{strain}, which is always positive, and the aromatic stabilization energy *ASE*, which is positive for even N and negative for odd N:

$$\Delta E_{cyc}(N) = E_{strain}(N) + ASE(N) \tag{S3}$$

The average of two consecutive cyclization energies is:

$$\frac{\Delta E_{cyc}(N) + \Delta E_{cyc}(N+1)}{2} = \frac{E_{strain}(N) + E_{strain}(N+1)}{2} + \frac{ASE(N) + ASE(N+1)}{2} \tag{S4}$$

The first term on the RHS of eq. (2) of two consecutive cyclization energies is the approximate strain energy in the vicinity of $N + 0.5$. The second term will be close to zero, as the two *ASE* values will have comparable magnitude and opposite sign. Therefore:

$$\frac{\Delta E_{cyc}(N) + \Delta E_{cyc}(N+1)}{2} \approx E_{strain}\left(N + \frac{1}{2}\right) \tag{S5}$$

The strain energy at N can then be evaluated as:

$$E_{strain}(N) \approx \frac{E_{strain}\left(N - \frac{1}{2}\right) + E_{strain}\left(N + \frac{1}{2}\right)}{2} \approx \frac{\Delta E_{cyc}(N)}{2} + \frac{\Delta E_{cyc}(N-1) + \Delta E_{cyc}(N+1)}{4} \tag{S6}$$

Combining eqs. (S6) and (S3), we obtain:

$$ASE(N) \approx \frac{\Delta E_{cyc}(N)}{2} - \frac{\Delta E_{cyc}(N-1) + \Delta E_{cyc}(N+1)}{4} \tag{S7}$$

which is equivalent to eq. (2) from main text reference 45.

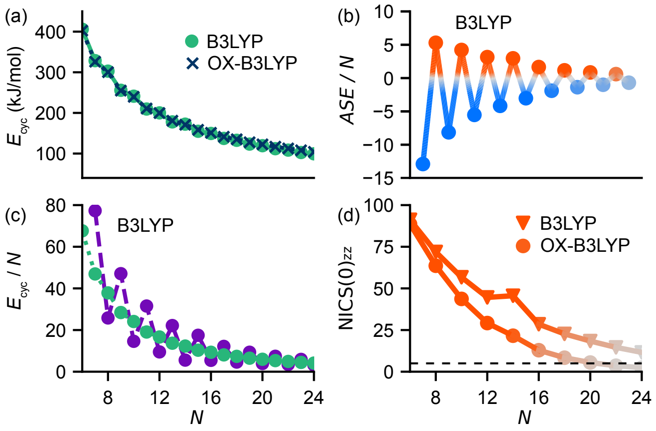

Figure S10. Cyclization energy (E_{cyc}), aromatic stabilization energies (*ASE*) and NICS(0)$_{zz}$ in in ***c*-P*N*** nanobelts with N = 6–24. (a) Cyclization energy (in kJ/mol). (b) Cyclization energy per porphyrin (green) and energy change associated with increasing the belt size from N to N+1 (purple). (c) *ASE* per porphyrin and (d) NICS(0)$_{zz}$ at different belt sizes.

(G) Other supplementary figures.

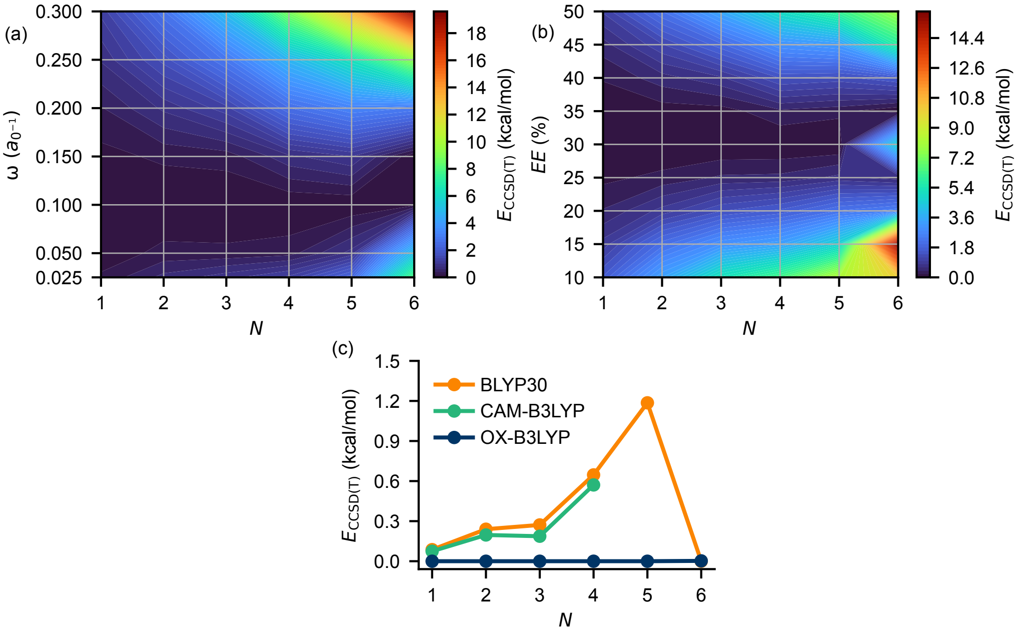

Figure S11. Heatmaps and plot illustrating CCSD(T) energies for edge-fused nanoribbons ***l*-P*N*** (N = 1–6). (a) Heatmap of CCSD(T) energies for different EE percentages in BLYPxx as a function of N. (b) Heatmap of CCSD(T) energies for different ω values in OX-B3LYP as a function of N. (c) Plot of the relative CCSD(T) energy for three DFAs (BLYP30, CAM-B3LYP, and OX-B3LYP) as a function of N.

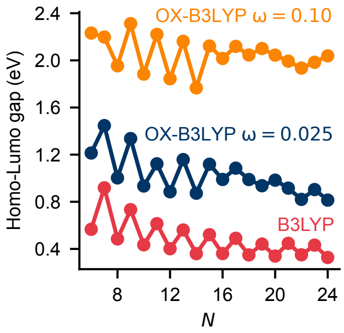

Figure S12. HOMO-LUMO gaps for ***c*-P*N*** nanobelts with N = 6–24, calculated using B3LYP, OX-B3LYP, and a variant of OX-B3LYP (ω = 0.10 a_0^{-1}).

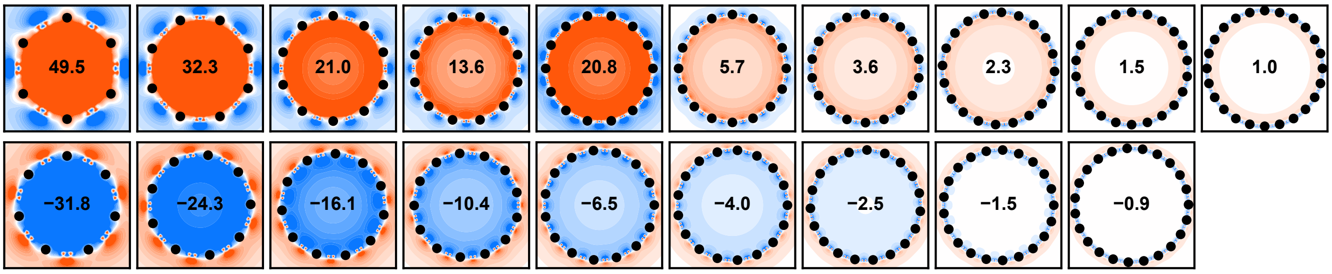

Figure S13. NICS(0)$_{zz}$ maps for ***c*-P*N*** nanobelts with N = 6–24, computed using BLYP30. The NICS(0)$_{zz}$ value in the center of the ring is shown in bold.

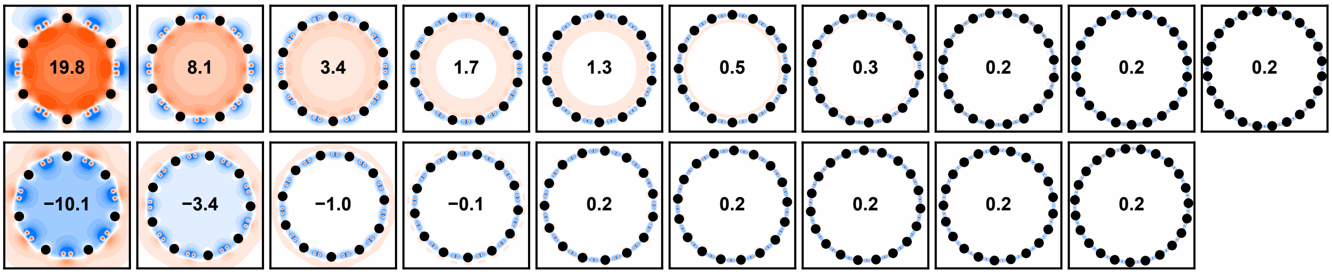

Figure S14. NICS(0)$_{zz}$ maps for ***c*-P*N*** nanobelts with N = 6–24, computed using a variant of OX-B3LYP (ω = 0.10 a_0^{-1}). The NICS(0)$_{zz}$ value in the center of the ring is shown in bold.

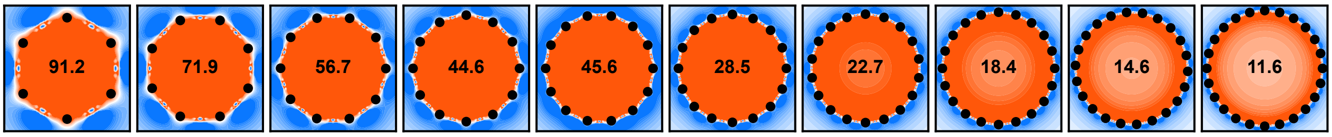

Figure S15. NICS(0)$_{zz}$ maps for ***c*-P*N*** nanobelts with even N = 6–24, computed using B3LYP. The NICS(0)$_{zz}$ value in the center of the ring is shown in bold.

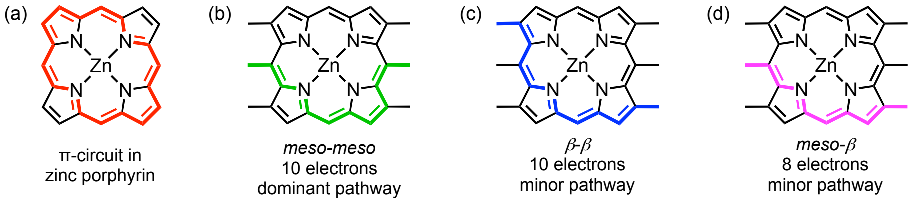

Figure S16. π-Circuits in porphyrin-based systems. (a) The π-circuit in zinc porphyrin (red).[15] (b) The dominant *meso-meso* pathways in edge-fused porphyrin systems (green). (c) The *β-β* pathway (blue) and (d) the *meso-β* pathway in edge-fused porphyrin systems. (For discussion of these conjugation pathways, see main text refs 16 and 49).

(H) Representative input files.

Section H1:

```
# Geometry optimizations with BLYP30 in Gaussian16

%nproc=36
%mem=72GB
#p opt def2svp BHandHLYP IOp(3/76=1000003000,3/77=0700007000)

Comment

0 1
@geom.xyz !geom.xyz must be devoid of comments and blank lines
```

```
# Geometry optimizations with CAM-BLYP (ω=0.10) in Gaussian16

%nproc=36
%mem=72GB
#p opt def2svp cam-b3lyp IOp(3/107=0100000000,3/108=0100000000)

Comment

0 1
@geom.xyz !geom.xyz must be devoid of comments and blank lines
```

```
# Geometry optimizations with a variant of OX-BLYP (ω=0.10, Ex^HF: 19% => 100%) in Gaussian16

%nproc=36
%mem=72GB
#p opt def2svp cam-b3lyp
IOp(3/107=0100000000,3/108=0100000000,3/119=0810000000,3/120=0810000000,3/130=01900,3/131=01900)

Comment

0 1
@geom.xyz !geom.xyz must be devoid of comments and blank lines
```

Section H2:

```
# Single point energy calculation with CCSD(T) in Orca

! DLPNO-CCSD(T) RIJCOSX cc-pVTZ cc-pVTZ/C

%maxcore 28000
%pal
    nprocs 32
end
* xyzfile 0 1 geom.xyz
```

```
# Single point energy calculation with SCS-MP2 in Orca

! DLPNO-SCS-MP2 RIJCOSX def2-TZVP def2/J def2-TZVP/C TightSCF

%MaxCore 28000
%pal
 nprocs 32
end
* xyzfile 0 1 geom.xyz
```

```
# Single point energy calculation with DSD-PBEP86-2013 D3BJ in Orca

! DLPNO-DSD-PBEP86/2013 D3BJ RIJCOSX def2-TZVP def2/J def2-TZVP/C TightSCF

%MaxCore 28000
%pal
 nprocs 32
end
* xyzfile 0 1 geom.xyz
```

DLPNO-SCS-MP2 calculations in Turbomole were done using the “define” options in following card:

```
# Define input options for single point energy calculation with SCS-MP2 in Turbomole
# prepare calculation by executing: define < input_options.txt


fpcc
a coord
*
no
b
all def2-TZVP
*
eht
y
0
y
ri
m 3500
on
jbas
b
all def2-TZVP
*
*
scf
conv
8
iter
100

pnocc
freeze
*
memory
28000
cbas
b
all def2-TZVP
*
pnoccsd
tolpno 3.33e-7
mp2
*
jkbas
b
all def2-TZVP
*
*
*
```

Section H3:

```
# Iterative energy calculation with CCSD(T1) in Orca

! DLPNO-CCSD(T1)-F12D TightPNO RIJCOSX cc-pVDZ-F12 cc-pVDZ-F12-CABS cc-pVTZ/C

%basis
newgto Zn "cc-pVTZ" end # Assign basis set to Zn
NewCABSGTO Zn "aug-cc-pVDZ-PP-OptRI" end # Assign CABS basis set to Zn
end
%maxcore 28000
%pal
 nprocs 32
end
* xyzfile 0 1 geom.xyz
```

Section H4:

```
# NICS with BLYP30 in Gaussian16

%nproc=36
%mem=180GB
#p nmr=giao geom=connectivity bhandhlyp def2svp IOp(3/76=1000003000,3/77=0700007000)

Comment

0 1
@geom.xyz !geom.xyz must be devoid of comments and blank lines

@connectivity
```

Section H5:

```
# tddft within RPA framework with BLYP30 in Orca

! BHANDHLYP def2-SVP def2-SVP/C def2/J RIJCOSX TightSCF

%method
    ACM 0.30, 0.70, 1.0
end
%maxcore 3500
%pal
    nprocs 36
end
* xyzfile 0 1 geom.xyz
%tddft
    nroots 20
    maxdim 20
    maxiter 250
    maxcore 3500
    tda false
end
```

```
# tddft within RPA framework with OX-BLYP (ω=0.10, Ex^HF: 19% => 100%) in Orca

! CAM-B3LYP def2-SVP def2-SVP/C def2/J RIJCOSX TightSCF

%method
    RangeSepEXX True
    RangeSepMu 0.10
    RangeSepScal 0.81 # should sum to 1 with ACM-A and ACM-B
```

```
    ACM 0.19, 0.00, 0.81 # ACM-A, ACM-B, ACM-C
end

%maxcore 28000

%pal
    nprocs 32
end

* xyzfile 0 1 geom.xyz

%tddft
    nroots 20
    maxdim 20
    maxiter 250
    maxcore 28000
    tda false
end
```

Section H6:

```
# molecular dynamics framework in xTB

$constrain
   elements: Zn
   force constant=0.0533
$end

$cma

$md
   temp= 298.15
   time= 10
   dump= 2
   step= 2
   velo= false
   nvt = true
   hmass= 4
   shake= 1
   sccacc= 2.0
   forcewrrestart= true
$end
```

References:

(1) F. Weigend, R. Ahlrichs, *Physical Chemistry Chemical Physics* **2005**, *7*, 3297–3305.

(2) M. J. Frisch, G. W. Trucks, H. B. Schlegel, G. E. Scuseria, M. A. Robb, J. R. Cheeseman, G. Scalmani, V. Barone, G. A. Petersson, H. Nakatsuji, X. Li, M. Caricato, A. V. Marenich, J. Bloino, B. G. Janesko, R. Gomperts, B. Mennucci, H. P. Hratchian, J. V. Ortiz, A. F. Izmaylov, J. L. Sonnenberg, Williams, F. Ding, F. Lipparini, F. Egidi, J. Goings, B. Peng, A. Petrone, T. Henderson, D. Ranasinghe, V. G. Zakrzewski, J. Gao, N. Rega, G. Zheng, W. Liang, M. Hada, M. Ehara, K. Toyota, R. Fukuda, J. Hasegawa, M. Ishida, T. Nakajima, Y. Honda, O. Kitao, H. Nakai, T. Vreven, K. Throssell, J. A. Montgomery Jr., J. E. Peralta, F. Ogliaro, M. J. Bearpark, J. J. Heyd, E. N. Brothers, K. N. Kudin, V. N. Staroverov, T. A. Keith, R. Kobayashi, J. Normand, K. Raghavachari, A. P. Rendell, J. C. Burant, S. S. Iyengar, J. Tomasi, M. Cossi, J. M. Millam, M. Klene, C. Adamo, R. Cammi, J. W. Ochterski, R. L. Martin, K. Morokuma, O. Farkas, J. B. Foresman, D. J. Fox, Wallingford, CT, **2016**.

(3) C. Riplinger, F. Neese, *The Journal of Chemical Physics* **2013**, *138*.

(4) S. Grimme, *J. Chem. Phys.* **2003**, *118*, 9095–9102.

(5) F. Neese, F. Wennmohs, U. Becker, C. Riplinger, *The Journal of Chemical Physics* **2020**, *152*, 224108.

(6) S. G. Balasubramani, G. P. Chen, S. Coriani, M. Diedenhofen, M. S. Frank, Y. J. Franzke, F. Furche, R. Grotjahn, M. E. Harding, C. Hättig, A. Hellweg, B. Helmich-Paris, C. Holzer, U. Huniar, M. Kaupp, A. Marefat Khah, S. Karbalaei Khani, T. Müller, F. Mack, B. D. Nguyen, S. M. Parker, E. Perlt, D. Rappoport, K. Reiter, S. Roy, M. Rückert, G. Schmitz, M. Sierka, E. Tapavicza, D. P. Tew, C. van Wüllen, V. K. Voora, F. Weigend, A. Wodyński, J. M. Yu, *The Journal of Chemical Physics* **2020**, *152*.

(7) L. Kong, F. A. Bischoff, E. F. Valeev, *Chemical Reviews* **2012**, *112*, 75–107.

(8) Z. Chen, C. S. Wannere, C. Corminboeuf, R. Puchta, P. v. R. Schleyer, *Chemical Reviews* **2005**, *105*, 3842–3888.

(9) R. Gershoni-Poranne, A. Stanger, *Chemical Society Reviews* **2015**, *44*, 6597–6615.

(10) D. Sundholm, H. Fliegl, R. J. F. Berger, *WIREs Comput. Mol. Sci.* **2016**, *6*, 639–678.

(11) A. Tsuda, A. Osuka, *Science* **2001**, *293*, 79–82.

(12) R. V. Belosludov, D. E. Nevonen, V. N. Nemykin, *J. Phys. Chem. A* **2021**, *125*, 2480–2491.

(13) C. Bannwarth, S. Ehlert, S. Grimme, *Journal of Chemical Theory and Computation* **2019**, *15*, 1652–1671.

(14) H. Zhu, Q. Chen, I. Rončević, K. E. Christensen, H. L. Anderson, *Angew. Chem. Int. Ed.* **2023**, *62*, e202307035.

(15) T. Tanaka, A. Osuka, *Chem. Rev.* **2017**, *117*, 2584–2640.
